# Development of drugs for severe malaria in children

**DOI:** 10.1093/inthealth/ihw038

**Published:** 2016-09-27

**Authors:** Phaik Yeong Cheah, Michael Parker, Arjen M. Dondorp

**Affiliations:** a Mahidol Oxford Tropical Medicine Research Unit, Faculty of Tropical Medicine, Mahidol University, 420/6 Rajvithi Rd, Bangkok, 10400, Thailand; b Centre for Tropical Medicine and Global Health, Nuffield Department of Clinical Medicine, University of Oxford, Oxford, UK; c The Ethox Centre, Nuffield Department of Population Health, University of Oxford, Oxford, UK

**Keywords:** Children, Clinical trial, Ethics, Mortality, Paediatrics, Severe malaria

## Abstract

Over 90% of deaths attributable to malaria are in African children under 5 years old. Yet, new treatments are often tested primarily in adult patients and extrapolations have proven to be sometimes invalid, especially in dosing regimens. For studies in severe malaria an additional complication is that the decline in severe malaria in adult patients precludes sufficiently powered trials in adults, before the intervention can be tested in the ultimate target group, paediatric severe malaria. In this paper we propose an alternative pathway to the development of drugs for use in paediatric severe malaria. We argue that following the classical phase I and II studies, small safety and efficacy studies using well-chosen surrogate endpoints in adult severe malaria be conducted, instead of larger mortality endpoint trials. If the drug appears safe and promising small pilot studies in paediatric severe malaria using the same endpoints can follow. Finally, with carefully observed safeguards in place to ensure high ethical standards, promising candidate interventions can be taken forward into mortality endpoint, well-powered, large paediatric studies in African children with severe malaria. Given the available research capacity, limited numbers of prudently selected interventions can be studied in phase III trials, and adaptive designs should be considered.

## Introduction

In contrast with all other endemic areas, transmission of falciparum malaria in sub-Saharan Africa is moderate to high, resulting in partial immunity against severe disease in older children and adults. This means almost all severe disease is in young children. Over 90% of all malaria attributable deaths are in African children under 5, amounting to about half a million deaths annually, despite recent gains in malaria control and efforts to increase pre-referral treatment.^1–4^ The need for additional drugs and interventions to improve survival in paediatric severe malaria is widely acknowledged.

The clinical presentation of severe malaria is different between adults and children, with more severe anaemia, convulsions and hypoglycaemia in children, and more severe jaundice, renal failure and pulmonary edema in adult patients. However, the presenting symptoms with most prognostic significance, which are coma depth and severity of metabolic acidosis, have very similar prevalence between adult and paediatric cases.^5^ Nevertheless, testing of new treatments in the main target group of African children is important, since extrapolation of research findings obtained in adults to children can be problematic. For instance, the antimalarial drugs sulphadoxine-pyramethamine and dihydroartemisinin-piperaquine have been introduced at too low doses in children causing unnecessary treatment failure, which has likely contributed to the development of drug resistance to the latter.^6,7^ It is thus clear that testing of new treatments in the main target group of African children is important.

Standard practice for drug development for severe falciparum malaria begins with initial phase I testing in healthy adults, mainly focusing on safety and pharmacokinetics. This phase is followed by phase II studies in adults with uncomplicated malaria, designed primarily for dose finding, safety and initial pharmacokinetic and pharmacodynamic evaluation. These are followed by larger phase III efficacy studies involving several thousand patients, usually performed in adult severe malaria outside Africa. Studies in the ultimate target group of African children under 5 years who have severe malaria are generally only done at the end of this development pathway.

The declining incidence of adult severe malaria means that large phase III studies in adults are increasingly problematic. To take one example, the SEAQUAMAT trial, which recruited 1259 adult participants in two years (from 2003 to 2005) when malaria incidence was much higher,^8^ would, with the current incidence, require 6 years. This problem jeopardises the development of much needed new treatments for severe malaria in African children. In this paper, we propose an alternative pathway to studying new interventions for paediatric severe malaria.

## A possible alternative pathway

Dwindling numbers of adults with severe malaria suggest the need to consider alternative intermediate groups, who are less vulnerable than small children, as study targets in drug development. However, none of the available groups—defined below according to age and the continuum in disease severity from uncomplicated, through moderately severe, to severe and lethal malaria—are suitable for this role.

Uncomplicated malaria in children: these children can be treated very effectively with an artemisinin combination therapy (ACT). Because ACTs are very efficacious, there is no additional benefit expected from additional therapies, which renders this group unacceptable to study.

Severe malaria in adolescents: this faces similar problems as the adult patient group, including extrapolation of results to children under 5 and challenges meeting the sample size.

Moderately severe malaria in adults: like uncomplicated malaria, this can be treated effectively with an ACT with no anticipated direct benefits from the new treatment. Moreover, even with a well-defined risk profile of the drug in this group, corresponding risks in children will be difficult to predict.

The absence of a suitable intermediate group suggests the need for an alternative approach. One such approach might be as follows:

Following the classical phase I and II studies, small safety and efficacy studies using well-chosen surrogate endpoints could be conducted in adults with severe malaria, instead of large mortality endpoint studies. The choice of surrogate endpoints will not only depend on their association with mortality, such as depth of coma and severity of prostration, but also on the pathophysiological target of the new intervention. For instance, adjuvant treatments aiming to improve microcirculatory flow could use the improvement in lactic acidosis as a surrogate endpoint for mortality, although its validity will have to be assessed more formally first.^9,10^ An intervention aiming at improving renal function can have the change in renal function as the primary endpoint.

If a new compound appears safe in adults, initial safety data in children might then be obtained from two sources. The first is where a related compound has been used extensively in the paediatric population. For example the safety of sevuparin, which is a heparinlike compound, can be extrapolated from heparin to a certain extent.^11^ A second source might be where data are available on the same compound used in different paediatric diseases. One example is inhaled nitric oxide, currently trialed in African children with cerebral malaria, and which has been studied previously in children with sickle cell crisis.^12^

Once sufficient prior safety data has been obtained, this could provide a rationale to conduct small pilot studies in children with severe malaria focusing on safety and using surrogate efficacy endpoints.

Finally, assuming an acceptable safety profile and a strong suggestion for benefit demonstrated by improvement in surrogate endpoints, a large-scale multi-centre, multi-country study in children with severe malaria with a mortality endpoint (or other appropriate endpoints) could be considered.

## Discussion

This paper proposes an alternative drug development pathway that does not involve conducting large mortality endpoint trials in adults with severe malaria as a prelude to paediatric trials.

It is important to note that the proposed strategy does not abandon the important principle that research should be conducted in the least vulnerable relevant group before moving on to more vulnerable groups. The proposal does not suggest bypassing studies in adult severe malaria, although there is precedence for such studies, for example the RTS,S/AS01 malaria vaccine trials in Africa.^13,14^

What are the strengths and limitations regarding the use of the proposed pathway?

### Strengths

The proposed pathway may lead to more effective development of new drugs, adjuvant therapies and supportive treatments for paediatric severe malaria. Studies are conducted in the main target group instead of being extrapolated from adult studies. It is time efficient and reduces unnecessary risks to patient groups to whom the exposure may not be necessary.

Recent international guidelines involving research with children have made a case for the protection of children through research rather than only from research.^15–18^ These guidelines stress that whilst conducting research involving children is complicated, this should not excuse inaction. Excluding children means depriving them from evidence-based medical care, increasing their vulnerability and, in the case of severe malaria, continued high mortality.

### Limitations

The risk-benefit balance for the proposed pathway might not be favourable for all possible scenarios. Omitting large trials in adults prior to studies in children cannot always fully exclude unexpected risks for the paediatric population. The Fluid Expansion as Supportive Therapy (FEAST) trial was a large randomised trial on fluid bolus therapy in African children with compensated septic shock, of which 57% had severe malaria.^13^ Pathophysiological studies and small trials in African children with severe malaria strongly suggested a benefit from fluid bolus therapy. The results of the FEAST trial showed, quite unexpectedly, a detrimental effect.

In addition, the use of variables as surrogate endpoints for mortality can have limitations. As a prerequisite, these variables, and normalisation of such variables, for example plasma lactate, should have been shown to correlate closely with mortality, and should be relevant in the pathophysiological chain of events causing death. The requirement that the variable used as a surrogate should have direct benefit for the patients is difficult to generalise. For instance, metabolic acidosis is difficult to translate to a direct discomfort for the patients, although it will cause shortness of breath. Faster recovery of coma has a clear direct benefit for the patient, but in contrast with metabolic acidosis, has no correlation with improved survival in large mortality endpoint severe malaria antimalarial drug trials.^3,8,19^

Another possible drawback is that subtle neurological side effects may be more difficult to discern in small children compared to adults, since they may not be able to express certain complaints, such as nightmares. In addition, specific adverse events might be more prominent in children, as seen with hypoglycaemia after treatment with the antimalarial drug, quinine. It is also possible that adverse events in young children may not become apparent until later in life. Neurocognitive development of children may have to be followed up years after the study has been completed. Finally, extrapolation of safety data from paediatric studies of similar compounds or from studies of the investigational product in other paediatric diseases is not always warranted, since there can be disease specific factors. An example is the use of heparin-like substances, tested in children with sickle cell crisis. Bleeding risks could be higher in severe malaria because of the invariable presence of thrombocytopenia.

### Ethical concerns and safe guards

Taken together, these considerations suggest that following careful scientific and ethical review, and the putting in place of measures to ensure high ethical standards, the alternative pathway outlined above may offer an acceptable route for the development of new drugs or other interventions in severe paediatric malaria.

It is acknowledged that including children earlier in the drug development process could pose ethical concerns, and assessment of risk benefit balance will remain crucial. This assessment will be more difficult if an intervention has not been used widely yet in adults.

Another important concern is the validity of the consent provided by parents or guardians. In the case of a child with severe malaria, the parent or guardian may find themselves in a position of ‘situational incapacity’ where their usual capacity to make these important decisions is compromised by the extreme stress of the situation, the time-critical nature of the intervention and the condition of their child. In some trials, the distress of the parents may be further compounded by the uncertainty of long term side effects and difficult-to-comprehend surrogate endpoints.

Additional safeguards should be in place, such as support for parents to make informed decisions on participation of their children in the study. For most trials, it will be important that interim analyses are conducted by an independent committee focusing on relevant safety endpoints in which the safety threshold for stopping the study should be clear and low. Study teams would need to ensure the availability of the best supportive care, which includes nursing care, optimum fluid therapy and setting-adapted intensive care.

Because of limited time and research capacity, innovative adaptive designs rather than conventional randomised controlled trials can be considered, which could accommodate testing several interventions in parallel.^20^ These designs allow interventions in under-performing study arms be dropped in an early stage.

## Conclusion

Despite important progress, mortality from severe malaria remains unacceptably high. There is an urgent need for research on new drugs and additional interventions in the main target group of African children. This paper suggests an approach that does not include large mortality endpoint trials in adults as a prelude to paediatric studies, yet assures safety and quality of the assessments.
